# Antibiotic-driven intestinal dysbiosis in pediatric short bowel syndrome is associated with persistently altered microbiome functions and gut-derived bloodstream infections

**DOI:** 10.1080/19490976.2021.1940792

**Published:** 2021-07-15

**Authors:** Robert Thänert, Anna Thänert, Jocelyn Ou, Adam Bajinting, Carey-Ann D. Burnham, Holly J. Engelstad, Maria E. Tecos, I. Malick Ndao, Carla Hall-Moore, Colleen Rouggly-Nickless, Mike A. Carl, Deborah C. Rubin, Nicholas O. Davidson, Phillip I. Tarr, Barbara B. Warner, Gautam Dantas, Brad W. Warner

**Affiliations:** a The Edison Family Center for Genome Sciences and Systems Biology, Washington University School of Medicine, St. Louis, Missouri, USA; b Department of Pathology and Immunology, Washington University School of Medicine, St. Louis, Missouri, USA; c Division of Newborn Medicine, Department of Pediatrics, Washington University School of Medicine, St. Louis, Missouri, USA; d Division of Pediatric Surgery, Department of Surgery, Washington University School of Medicine, St. Louis, MO, USA; e Department of Medicine, Washington University School of Medicine, St. Louis, MO, USA; f Department of Molecular Microbiology, Washington University School of Medicine, St. Louis, Missouri, USA; g Division of Gastroenterology, Hepatology, and Nutrition, Department of Pediatrics, Washington University School of Medicine, St. Louis, Missouri, USA; h Department of Biomedical Engineering, Washington University in St. Louis, St. Louis, Missouri, USA

**Keywords:** Short bowel syndrome, bloodstream infections, antibiotics, microbiota, intestinal dysbiosis, shotgun metagenomics, functional profiling, strain-tracking

## Abstract

Surgical removal of the intestine, lifesaving in catastrophic gastrointestinal disorders of infancy, can result in a form of intestinal failure known as short bowel syndrome (SBS). Bloodstream infections (BSIs) are a major challenge in pediatric SBS management. BSIs require frequent antibiotic therapy, with ill-defined consequences for the gut microbiome and childhood health. Here, we combine serial stool collection, shotgun metagenomic sequencing, multivariate statistics and genome-resolved strain-tracking in a cohort of 19 patients with surgically-induced SBS to show that antibiotic-driven intestinal dysbiosis in SBS enriches for persistent intestinal colonization with BSI causative pathogens in SBS. Comparing the gut microbiome composition of SBS patients over the first 4 years of life to 19 age-matched term and 18 preterm controls, we find that SBS gut microbiota diversity and composition was persistently altered compared to controls. Commensals including *Ruminococcus, Bifidobacterium, Eubacterium*, and *Clostridium* species were depleted in SBS, while pathobionts (*Enterococcus*) were enriched. Integrating clinical covariates with gut microbiome composition in pediatric SBS, we identified dietary and antibiotic exposures as the main drivers of these alterations. Moreover, antibiotic resistance genes, specifically broad-spectrum efflux pumps, were at a higher abundance in SBS, while putatively beneficial microbiota functions, including amino acid and vitamin biosynthesis, were depleted. Moreover, using strain-tracking we found that the SBS gut microbiome harbors BSI causing pathogens, which can persist intestinally throughout the first years of life. The association between antibiotic-driven gut dysbiosis and enrichment of intestinal pathobionts isolated from BSI suggests that antibiotic treatment may predispose SBS patients to infection. Persistence of pathobionts and depletion of beneficial microbiota and functionalities in SBS highlights the need for microbiota-targeted interventions to prevent infection and facilitate intestinal adaptation.

## Introduction

Surgical removal of the intestine is often necessary to treat gastrointestinal disorders such as necrotizing enterocolitis, volvulus, gastroschisis, and intestinal atresia.^1^ Extensive intestinal loss may result in a form of intestinal failure known as short bowel syndrome (SBS).^2,3^ In SBS, reduced intestinal surface area is inadequate for normal nutrients, electrolytes, and fluid absorption. As a result, patients require sustained parenteral nutrition (PN) to support growth and development.^4^ While lifesaving, prolonged PN is associated with SBS-related morbidity and mortality, most notably bloodstream infections (BSIs)^1,5^ and PN-associated liver disease (PNALD).^2,6^ Further, SBS patients frequently develop increased bowel caliber and reduced peristalsis resulting in small bowel bacterial overgrowth (SBBO).^7^ Increased gut-derived bacterial burden in the context of SBBO is implicated in contributing to PNALD.^8^ Retrospective studies of BSIs in SBS patients have further implicated common constituents of the intestinal flora.^9^ As a result, SBS patients are frequently exposed to multiple courses of oral and intravenous antibiotics to prevent and treat SBBO, PNALD, and BSI.

Diversity and richness of the gut microbiota (GM) in children with SBS are lower than corresponding indices in age-matched controls.^10–12^ The SBS GM is characterized by increased abundance of *Enterobacteriaceae* and depletion of short-chain fatty acid-producing obligate anaerobes,^10,11,13^ an imbalance that has been associated with poor growth.^14^ While prior work has identified the GM as a determinant of successful weaning from PN, conclusions are limited by sample size, lack of suitable age-matched preterm and term controls, and cross-sectional nature of comparisons. Also, most studies have relied on 16*S* ribosomal RNA (rRNA) gene sequencing, which does not assess the functional consequences of microbial dysbiosis and hinders identification of the main drivers of SBS GM development.

Genome-resolved metagenomic profiling in longitudinal studies offers deeper insights into the long-term functional microbiome consequences associated with SBS and can inform nutrition management. Time-series study of the GM is critical, as children with SBS are frequently exposed to broad-spectrum antibiotics throughout childhood.^15^ Each exposure can abruptly and persistently alter the GM, enrich the pool of antibiotic resistance genes (ARGs), and select for pathobionts,^16,17^ which are constituents of the microbiota with increased pathogenic potential. Additionally, altered gut physiology may predispose to pathogen translocation into circulation, resulting in BSI. Indeed, the gut has been identified as a source for bacterial infections in premature infants,^18^ hematopoietic stem cell transplant patients,^19^ and patients who received fecal microbiota transplants.^20^ However, the risk associated with gastrointestinal pathobiont colonization and antibiotic exposure in SBS patients is unclear.

Here, we use multivariate statistics and deep metagenomic sequencing to show that repeated antibiotic exposures in early life hinder recovery of gut microbiota diversity and durably enrich for ARGs and pathobionts, while depleting beneficial commensals and microbiota functions. We hypothesized that intestinal pathobiont enrichment in SBS may serve as a source for BSI. Pairing deep metagenomic sequencing of the intestinal microbiota with isolate sequencing of BSI isolates, we provide genome-resolved evidence that gut-residing pathobionts cause repeated BSIs in SBS patients throughout the first 4 years of life.

## Results

### SBS patient cohort and controls

To test the hypothesis that taxa and functions of the GM vary from normal development throughout early life in SBS, we first analyzed 159 stools, collected over the first four years of life from 19 children with SBS (*n* = 19) and 37 age-matched term (*n* = 19) and preterm (*n* = 18) controls using whole metagenome shotgun sequencing (Table 1). Preterm infant samples included in this study were collected during the first 2 years of life. One SBS participant had a higher age at sampling compared to all other infants (SBS 05) and was therefore excluded from comparative analysis.

**Table 1.** Cohort overview

		SBS	Term	Preterm
Participants		19	19	18
Sex	Female	7 (36.8%)	9 (47.4%)	10 (55.6%)
	Male	12 (63.2%)	10 (52.6%)	8 (44.4%)
Gestational age [median (range)]		34 (24–39)	36 (33–37)	26 (23–28)
Bowel remaining (cm) [median (IQR)]		36.75 (25.63–58.5)	-	-
Ileocecal Valve	Present	5 (26.3%)	-	-
Samples [n]		57	47	55
DOL at sampling [median (IQR)]		561 (394–867)	451 (298–623)	279 (79–422)
Read depth (million) [median (IQR)]		5.17 (3.79–6.37)	4.83 (4.00–6.41)	4.30 (3.30–5.13)

*SBS = Short bowel syndrome

### The intestinal microbiota in SBS are persistently altered throughout early life

**Figure 1.**
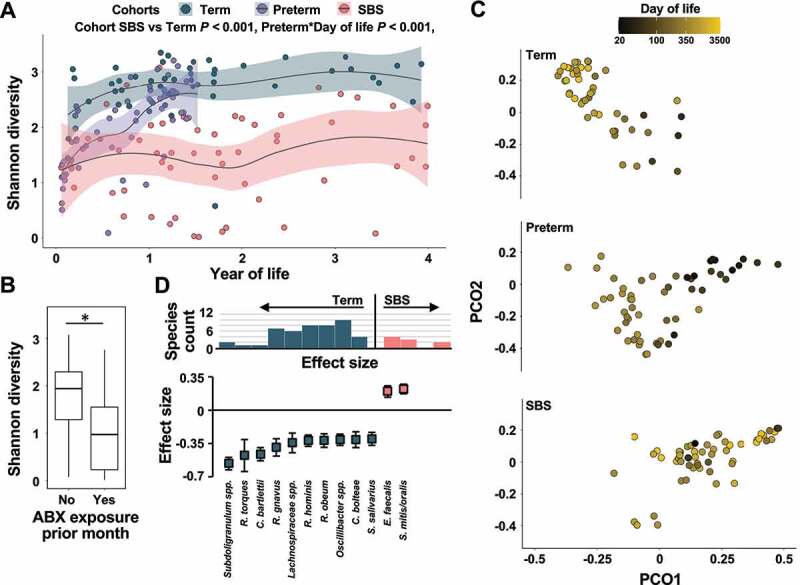
**Taxonomic composition of the SBS microbiota compared to preterm and term controls**. (a) Shannon diversity indices of the gut microbiota of SBS patients (salmon), preterm (purple) and term controls (teal) by year of life (*n* = 159). Loess regression lines with 95% confidence interval shading are drawn. All *P-*values are two-tailed, from longitudinal maximum-likelihood GLMMs Tukey-adjusted for multiple comparisons. (b) Boxplot of Shannon diversity of SBS gut microbiota with or without exposure to antibiotics in the month prior to sampling (*n* = 51, *P* = .042, longitudinal maximum-likelihood GLMM FDR corrected). (c) Principal Coordinate Analysis (PCoA) plot of species based on the Bray–Curtis dissimilarity index for all samples (*n* = 159), colored by day of life. (d) Species enriched (salmon) or depleted (teal) in SBS patients compared to term controls as determined via MaAsLin2. (Top) Number of species significantly depleted or enriched in SBS or term controls plotted against determined binned effect sizes. (Bottom) Top 20% of species depleted or enriched in SBS patients compared to term controls selected based on determined effect size

Longitudinal GLMMs for microbiota taxonomic alpha (Shannon) diversity demonstrated that children with SBS had lower alpha diversity than did term children throughout the first four years of life (*n* = 104 samples, *P* = 1.76e^−6^, maximum-likelihood GLMM Tukey corrected for multiple comparisons, Figure 1a). However, they did not statistically differ from preterm infants during the first 2 years of life (*n* = 112 samples, *P* = .998, maximum-likelihood GLMM Tukey corrected for multiple comparisons, Figure 1a). While alpha diversity positively correlated with increasing day of life in the preterm cohort (*n* = 55, *P* = <0.001, maximum-likelihood GLMM), the GM in SBS infants did not show a similar trend (*n* = 57, *P* = .853, maximum-likelihood GLMM). To identify the main drivers of microbiota diversity throughout the first 4 years of life in SBS, we further constructed SBS-specific GLMMs incorporating clinical metadata (Methods, Supplementary Data Table 1). Antibiotic exposure in the prior month negatively correlated with microbiota alpha diversity (*n* = 49, *P* = .042, maximum-likelihood GLMM FDR corrected for multiple comparisons, Figure 1b), while male sex (*n* = 49, *P* = .483, maximum-likelihood GLMM FDR corrected for multiple comparisons) and presence of the ileocecal valve (*n* = 49, *P* = .456, maximum-likelihood GLMM FDR corrected for multiple comparisons) correlated with increased alpha diversity but were not significant after multiple hypothesis correction. Further, microbiota composition also has differed significantly among the SBS, preterm and term cohorts (*n* = 159, *P* = .002, repeat-measures PERMANOVA, Figure 1c). While the day of life accounted for a significant portion of the variance of the microbiota composition in both the preterm and term cohorts (*n* = 55 and *n* = 47, *P* = .001 and *P* = .001, respectively, repeat-measures PERMANOVA, Figure 1c), it did not have a similar effect in the SBS cohort (*n* = 57, *P* = .311, repeat-measures PERMANOVA, Table 2). Inter-individual variability accounted for the majority of variance in GM composition in SBS patients (*n* = 57, *P* = .001, repeat-measures PERMANOVA, Table 1), with current exposure to enteral nutrition and race having smaller, non-significant effects (*n* = 57, *P* = .072 and *P* = .061, repeat-measures PERMANOVA, Table 1). 57 and 21 species were identified to be significantly enriched or depleted in SBS compared to term or preterm infants, respectively (Figure 1d, Supplementary Figure 1, 2, Supplementary Data Tables 2, 3). Specifically, a variety of commensal *Ruminococcus, Bifidobacterium, Eubacterium*, and *Clostridium* species were depleted in SBS compared to both preterm and term controls, respectively (*n* = 104 and *n* = 112, respectively, *qval = *<0.05, MaAsLin2, Figure 1d, Supplementary Figure 1, 2). Conversely, multiple species with pathogenic potential, including typical gut bacteria such as *Enterococcus faecalis* as well as taxa not traditionally considered constituents of the intestinal microbiota such as *Staphylococcus aureus* or oral and respiratory *Streptococcus*, were significantly enriched in stools of the SBS cohort (*qval *= <0.05, MaAsLin2, Figure 1d, Supplementary Figure 1, 2).

**Table 2.** Variance of microbiota composition explained by clinical variables determined via repeat measures PERMANOVA

	Variation explained	*P*-value
**Gestational age at birth**	2.12	0.814
**Sex**	2.99	0.391
**Race**	4.45	*0.061*
**Ileocecal Valve (ICV)**	9.24	0.244
**Small bowel bacterial overgrowth (SBBO)**	5.62	0.466
**Current antibiotics**	4.15	0.71
**Antibiotics in prior month**	3.69	0.777
**Length of bowel remaining**	3.74	0.587
**Day of life**	2.12	0.311
**Current enteral nutrition**	5.46	*0.072*
**Enteral nutrition ever**	4.96	0.153
**Current parenteral nutrition**	6.32	0.444
**Height percentile**	2.38	0.137
**Weight percentile**	2.65	0.193
**Participant**	45.76	***0.001***
**ALL**	51.15	0.215

To assess whether microbiota signatures found in the first 4 years of life can also be observed later in life, we analyzed the intestinal microbiota of participant SBS 05 with samples collected in adolescence (average age at sampling 16.2 years). We observed similarly high abundances of pathobionts (*E. coli, K. pneumoniae*, Supplementary Figure 3), which was associated with low Shannon diversity values (<2), but not necessarily preceded by antibiotic exposure.

### The functional capacity of the SBS microbiome varies compared to healthy controls

**Figure 2.**
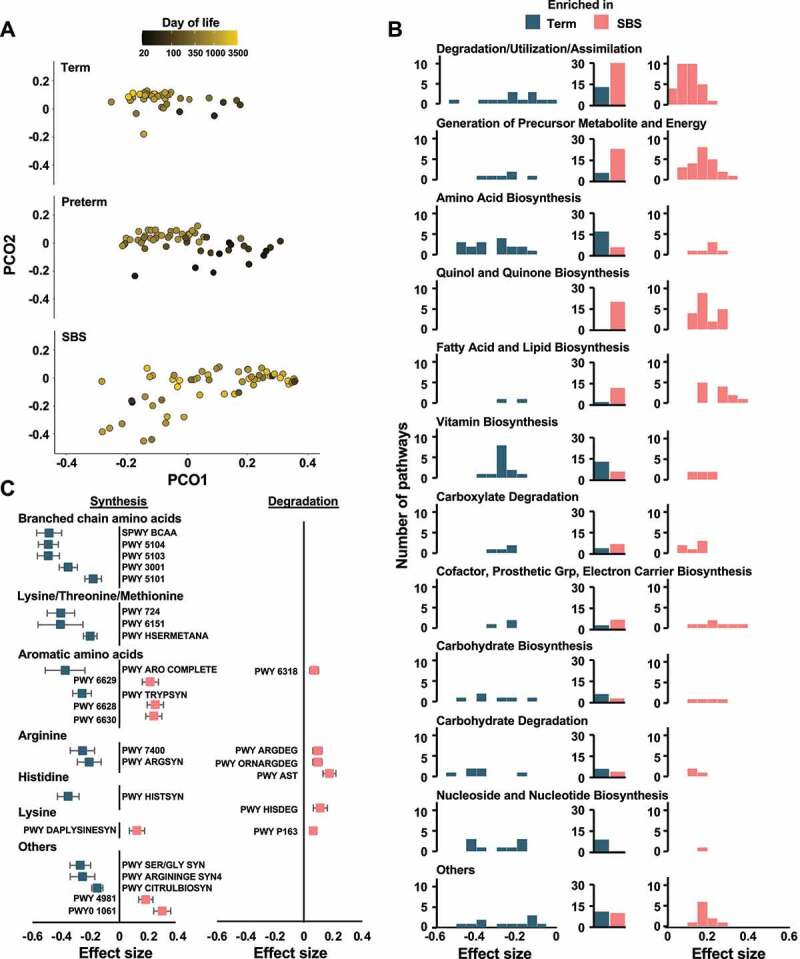
**Functional composition of the SBS microbiota throughout the first years of life**. (a) Principal Coordinate Analysis (PCoA) plot of functional pathway abundance based on the Bray–Curtis dissimilarity index for all samples (*n* = 159), colored by day of life. (b) Pathways significantly enriched (salmon) or depleted (teal) in SBS patients aggregated into functional categories based on MetaCyc hierarchy compared to term controls as determined via MaAsLin2. (Left and right) Number of pathways significantly depleted or enriched in SBS or term controls within each functional category plotted against determined binned effect sizes. (Middle) Sum of all depleted or enriched pathways within each functional category grouped by depletion and enrichment status in SBS patients compared to term controls. (c) Amino acid synthesis or degradation pathways significantly enriched (salmon) or depleted (teal) in SBS patients compared to term controls as determined via MaAsLin2

The GM serves critical functions for its host, including digesting complex nutritional components to provide energy-rich metabolites for healthy growth and development.^21^ Based on wide-ranging depletion of commensal species in SBS compared to both preterm and term controls, we hypothesized that key functional capabilities of the microbiota are persistently altered in SBS. Indeed, we identified significant changes in the functional profiles of the GM in SBS compared to preterm and term controls (*n* = 159, *P* < .01, repeat-measures PERMANOVA, Figure 2a). A significant fraction of the variance was explained by day of life in the preterm and term cohorts, but not in SBS children (*n* = 55, *n* = 47, and *n* = 57, *P* < .001, *P* < .001, and *P* = .137, respectively, repeat-measures PERMANOVA). We also have identified 199 metabolic pathways encoded by the microbiota persistently altered in SBS compared to term infants throughout the first 4 years of life (*n* = 104, *qval = *<0.05, MaAsLin2, Supplementary Data Table 4). Functional aggregation of significantly altered pathways indicated that pathways involved in quinol and quinone biosynthesis (*P* = <0.001, Fisher’s exact test, Benjamini–Hochberg corrected, Figure 2b) were significantly enriched in the metabolic profile of the SBS microbiota, while pathways involved in amino acid biosynthesis (*P* = .015, Fisher’s exact test, Benjamini-Hochberg corrected) and nucleoside and nucleotide biosynthesis (*P* = .02, Fisher’s exact test, Benjamini–Hochberg corrected) were significantly depleted. Specifically, pathways involved in branched chain amino acids, lysine, threonine, methionine, and histidine biosynthesis were depleted in the microbiota of SBS infants, while degradation pathways of various amino acids were enriched in the microbiome of SBS children (Figure 2c).

### The resistome in SBS is persistently altered and enriched for broad-spectrum ARGs

**Figure 3.**
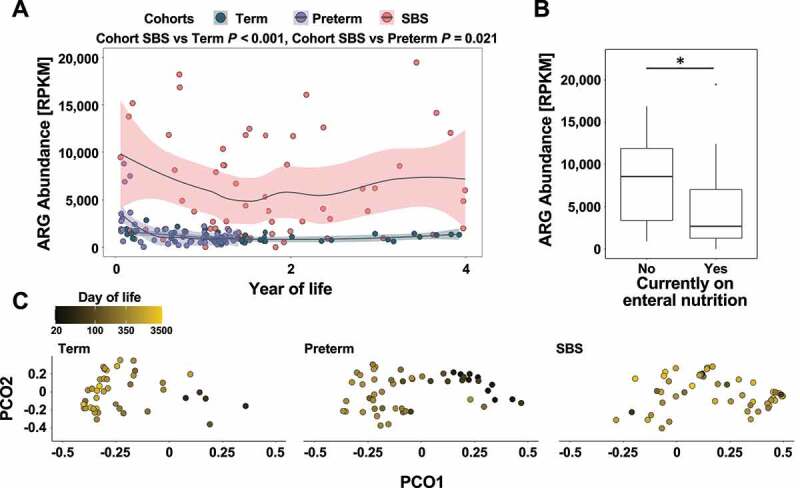
**Resistome composition in SBS throughout the first years of life**. (a) ARG abundance measured in RPKM within the microbiome of SBS patients (salmon), preterm (purple) and term controls (teal) by year of life (*n* = 159). Loess regression lines with 95% confidence interval shading are drawn. All *P-*values are two-tailed, from longitudinal maximum-likelihood GLMMs Tukey-adjusted for multiple comparisons. (b) Boxplot of ARG abundance measured in RPKM within the microbiome of SBS patients with or without exposure to enteral nutrition at sampling (*n* = 51, *P* = .011, longitudinal maximum-likelihood GLMM). (c) Principal Coordinate Analysis (PCoA) plot of ARG abundance profiles based on the Bray–Curtis dissimilarity index for all samples (*n* = 159), colored by day of life

SBS patients are exposed to antibiotics throughout childhood.^15^ As such exposures can enrich the intestinal reservoir for ARGs (the “resistome”),^16^ we hypothesized that the resistomes of SBS infants would be enriched compared to that of age-matched preterm and term controls. We found increased ARG abundance in the intestinal microbiomes of SBS infants throughout the first years of life compared to term and preterm infants, respectively (*n* = 104, and *n* = 112, *P* = <0.001 and *P* = .021, maximum-likelihood GLMM Tukey corrected for multiple comparisons, Figure 3a, Supplementary Data Table 5). Current exposure to enteral nutrition correlated with decreased ARG abundance (*n* = 51, *P* = .011, maximum-likelihood GLMM, Figure 3b). Conversely, resistome diversity (Shannon diversity) and ARG richness did not differ significantly between SBS patients and control cohorts (*P* > .895 for all comparisons, maximum-likelihood GLMM Tukey corrected for multiple comparisons, Supplementary Figure 4A, B). Resistome composition differed significantly between SBS patients and both preterm and term control cohorts throughout infancy (*n* = 159, *P = *.001, repeat-measures PERMANOVA, Figure 3c). Similar to the observed effect of day of life on the taxonomic composition of the GM, we found that age has explained a significant portion of the resistome variance in term and preterm children (*n* = 47 and *n* = 55, *P* = .001 and *P* = .001, respectively, repeat-measures PERMANOVA, Figure 1d), but not in SBS patients (*n* = 57, *P* = .453, repeat-measures PERMANOVA, Figure 3c). We aggregated ARGs by resistance class to further characterize resistome differences between cohorts. Abundance of four ARG classes correlated significantly with SBS or control status (*P* < .05, maximum-likelihood GLMM Tukey corrected for multiple comparisons, Supplementary Figures 5A-D). SBS status was positively correlated with the abundance of broad-spectrum efflux pump ARGs compared to the term cohort, while lincosamide ARGs were negatively correlated with SBS status. Resistance modulator ARGs were significantly more abundant, while fosfomycin ARGs were depleted in SBS compared to preterm infants.

### Persistent gut pathogens can cause recurrent bacteremia

**Figure 4.**
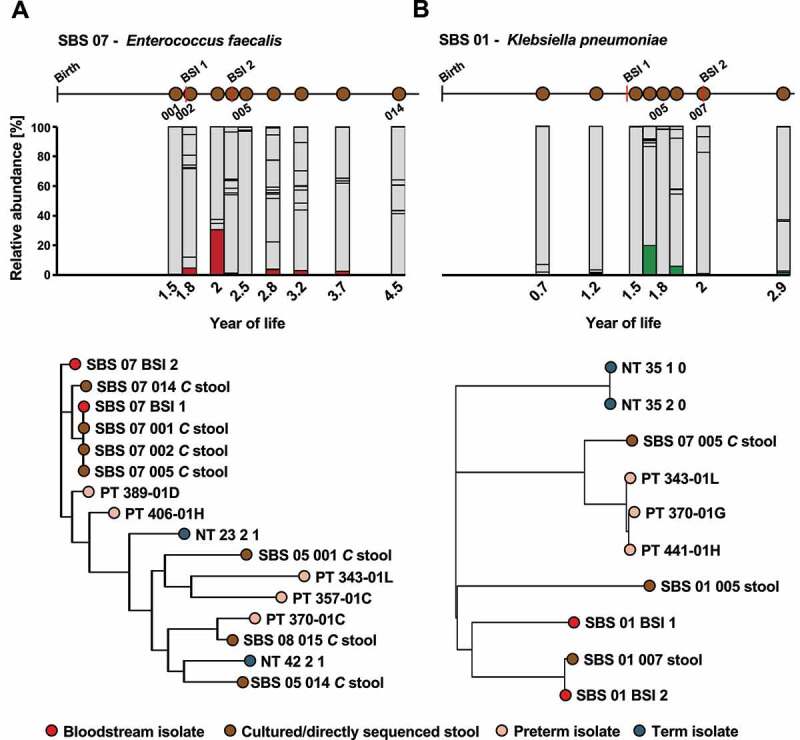
**Gut-persisting pathobionts can cause repeated episodes of BSI in SBS**. (a) *E. faecalis* persists in patient SBS 07 causing two BSI over 3 years of life. (Top) Relative abundance of *E. faecalis* in stools (red) by year of life. Other species are depicted in gray. Schematic of sample collection and BSI events is shown on top. (Bottom) Phylogenetic relatedness of BSI isolates and metagenomic strains based on core SNPs as assessed by StrainSifter. Branch tip colors indicate BSI isolates (red), SBS (brown) and preterm (peach), or term (teal) stools. (b) *K. pneumoniae* found in a stool of patient SBS 01 concurrently causes a BSI. (Top) Relative abundance of *K. pneumoniae* in stools (green) by year of life. Other species are depicted in gray. Schematic of sample collection and BSI events is shown on top. (Bottom) Phylogenetic relatedness of BSI isolates and metagenomic strains based on core SNPs as assessed by StrainSifter. Branch tip colors indicate BSI isolates (red), SBS (brown) and preterm (peach), or term (teal) stools

The gut is increasingly recognized as the pre-dissemination habitat of bacteria that cause BSI.^18–20^ As we observed enrichment of pathobionts in the intestinal tract of SBS patients, we hypothesized that intestinal pathobionts may cause bacteremia in patients with SBS. To investigate this hypothesis, we performed whole-genome sequencing of 13 BSI isolates from the SBS cohort (Supplementary Data Table 6). We compared the phylogenetic relatedness of BSI isolates to stool strains from corresponding patients, as well as unrelated terms and preterm infants recruited in the same hospital system. We identified 3 BSI isolates (two *E. faecalis* and 1 *K. pneumoniae*) that belonged to the same strain (1–6 SNPs and 128 SNPs, respectively, Supplementary Data Table 7) as species representatives recovered from stools (Figure 4a, b). Interestingly, in one participant (SBS 07), a strain of *E. faecalis* was found in the stool prior to any BSIs and persisted in the stool for ~2.7 years of subsequent sampling. While the *E. faecalis* abundance over this time remained low, averaging 4.99%, this strain caused two episodes of BSI separated by ~8.5 months (Figure 4a). Similarly, we found that a strain of *K. pneumoniae* causing a BSI in participant SBS 01 was found at the time of the infection in the gut (sample SBS 01 007). However, a second episode of BSI experienced by the participant ~7.7 months earlier was caused by an unrelated strain of *K. pneumoniae* and a third unrelated strain was found to dominate a previous stool specimen (Figure 4b, Supplementary Data Table 7).

## Discussion

Providing adequate nutrition for growth and ultimately achieving enteral autonomy by promoting intestinal adaptation are the goals of pediatric SBS management.^15^ Intestinal microbiota may contribute to intestinal adaptation either directly or by producing host-interactive metabolites that stimulate intestinal immune development and enterocyte proliferation.^22,23^ Prior studies have largely employed 16*S* rRNA gene sequencing, which allows taxonomic description, but is inadequate for characterizing the metabolic potential of the microbiota in SBS. Moreover, characterization of SBS microbiota has not been supported by longitudinal sampling with appropriate age-matched controls, a prerequisite for robust interrogation of the pediatric intestinal microbiome, which changes rapidly in the first years of life.^24^ Here, we mitigate these issues by analyzing a longitudinally-sampled cohort of pediatric SBS patients. We comprehensively evaluated taxonomic, functional, and resistome correlates of SBS by comparing them to two age-matched cohorts of term and preterm infants recruited in the same hospital system.

Consistent with previous reports,^10–12,14^ we find that GM diversity is decreased in SBS compared to term children and that this signature persists throughout the first four years of life. We identify antibiotic exposure as the main driver of diminished GM diversity in SBS. Interestingly, early life microbiota diversity between preterm and SBS infants did not differ significantly in our analysis. This finding may reflect the similarly high rate of antibiotic exposure early in life.^16^ However, while microbiota diversity increased with day of life in preterm infants with intact intestinal anatomy, an effect attributed to reduced antibiotic exposure after hospital discharge,^17^ such a correlation was not observed in SBS patients. Overall, our data suggest that pediatric SBS patients do not undergo GM maturation common to both term and preterm infants during the first two years of life.^24,25^ The decreased diversity and lack of GM maturation likely reflect that both hospitalizations and antibiotic exposures remain substantial beyond infancy in children with SBS, because of their increased risk of serious bacterial infections secondary to BSI and SBBO.^7^ This underscores the importance of continued studies on mechanisms of intestinal adaptation. Sampling of a 16-year-old SBS participant further suggests that this trend may continue into adolescence, an important question that should be investigated further.

Functional profiling of SBS microbiota has been limited by sample size and the availability of appropriate age-matched controls,^14^ but is critical to our understanding of the role of microbiota in intestinal adaptation and immune maturation.^23^ Here, we show that the altered taxonomic composition of the intestinal microbiota in SBS is accompanied by alterations of the encoded metabolic repertoire compared to healthy-term children. Key biosynthetic functions provided by healthy microbiota, including synthesis of vitamins, nucleosides/nucleotides, and amino acids were persistently depleted in pediatric SBS microbiota. Microbiota-derived B vitamins such as folate and thiamine play key roles in diverse processes, such as DNA methylation and intestinal adaptation.^23,26,27^ Similarly, the intestinal microbiota influences gut concentration of branched-chain amino acids, which are beneficial for gut homeostasis, intestinal immunity, barrier function and growth.^28–30^ Depletion of these key biosynthetic pathways in SBS may contribute to chronic malabsorption and thereby impaired growth.^15^ Conversely, enrichment of fatty acid and lipid biosynthetic pathways may contribute to colonic inflammation observed in SBS.^22,31^ We also found that intestinally-derived lipids enriched in the SBS cohort, including palmitate, lipopolysaccharide, and oleate, may contribute to intestinal inflammation and mucosal injury.^32–34^ Furthermore, we observed wide-ranging alterations of central microbiota functionalities, including enrichment of aerobic respiration, gluconeogenesis, glycolysis, TCA cycle, heme biosynthesis, and fermentation pathways, reflective of the altered physiological conditions in the SBS intestine. Further cohort studies are necessary to resolve the functional impact of these alterations on host physiology and their association with intestinal adaptation.

As antibiotic exposure shapes resistome enrichment and composition,^16,17^ we hypothesized that frequent antibiotic exposure in SBS patients would diversify and enrich the resistome. Indeed, we observed an increased abundance of ARGs and restructuring of the resistome composition in SBS patients compared to preterm and term cohorts. However, we did not find the resistome to be more diverse or enriched for a greater quantity of unique ARGs compared to the preterm and term cohorts. This indicates that an increased abundance of pathobionts, previously associated with increased ARG abundance,^35^ is likely the main determinant of the increased abundance of ARGs in SBS. Current enteral nutrition, associated with altered taxonomic composition of the SBS microbiota, was negatively correlated with ARG abundance, supporting the idea that taxonomy and resistome in SBS are connected in early life.

Intestinal pathobiont presence has been identified as a source for BSI in multiple vulnerable patient cohorts, including preterm infants.^18–20^ It has been hypothesized that the intestinal microbiota may similarly be the source of BSI common in SBS patients, as there is a greater abundance of pathobionts including *E. coli, E. faecalis*, and *K. pneumoniae* in their intestinal microbiota compared to healthy controls.^9,36^ Previous research has shown that the GM of SBS patients with BSI is marked by a significantly increased pathobiont abundance and lower alpha diversity compared to SBS patients without BSI.^11^ Here, we combine whole-genome sequencing of SBS patients’ BSI isolates and culture-enriched metagenomics of stool specimens to provide evidence that gut-derived pathobionts can cause repeated BSI throughout early life and persist for years despite SBBO prophylaxis and treatment with antimicrobial therapy. We found that these pathobionts are frequently minority constituents of the GM and further investigation is needed to determine if intestinal blooms prior to infectious episodes are required to facilitate bacterial translocation into the bloodstream, as observed in other gut-derived infections.^37^ Our demonstration of persistence of gut bacteria that cause recurrent episodes of BSIs suggests that gut colonization with bacteria with invasive potential may not be transient. Patients with clinical symptoms suggestive of SBBO are typically treated with antibiotics to suppress strict and facultative anaerobes,^38^ which may decrease chronic diarrhea and malabsorption. However, our study found other gut bacteria not typically targeted in SBBO treatment that caused BSI, possibly resulting from gut barrier dysfunction in SBS. Unfortunately, these bacteria are enriched in genes encoding resistance to parenteral antibiotics used for treatment and prophylaxis of BSI and SBBO. These findings highlight the complexity of decision-making surrounding antibiotic use in this patient population. Given the detrimental effects on microbiota composition and function, antibiotic stewardship is critical not only for optimizing intestinal adaptation in the pediatric SBS patient population, but also for decreasing risk of serious infections and antibiotic resistance.

Though it is the largest longitudinal sample collection from SBS patients reported to date, our study was still limited by sample availability. Thus, our finding that representatives of only 3/13 strains causing a BSI event could be trackedto the intestine may underestimate the true burden of gut-derived BSI events, as fecal samples collected within days of BSI onset were often unavailable. Future studies with more granular sampling schemes should aim to assess the burden of gut-derived BSI events quantitatively. The severity and consequences of surgical intervention in SBS can vary significantly between patients and success of intestinal adaptation in childhood is dependent on a multitude of factors, including remaining bowel length, nutritional status, bowel function, antibiotic exposure, and presence of ICV and colon.^15^ Given this variability in the pediatric SBS population, future studies must rely on large, well-defined patient cohorts to comprehensively address the impact of all potentially relevant factors on the intestinal microbiota and intestinal adaptation. Large, prospective cohort studies, similar to those reported for inflammatory bowel disease,^39^ combining high-resolution metabolomics, lipidomics, and metagenomics are warranted for detailing the role of the microbiota in post-surgical intestinal adaptation in SBS. Nonetheless, our findings support the important and enduring effect of intestinal taxonomic changes on metabolic pathways in SBS patients. Additionally, our finding of increased gut carriage of pathobionts and altered antibiotic resistance genes driven by antibiotic exposure underscore the necessity of judicious antibiotic use in this high-risk population.

## Methods

### Study design

Patients with SBS were recruited from the Pediatric Intestinal Rehabilitation Clinic at St. Louis Children’s Hospital (SLCH). SBS was defined as parenteral nutrition requirement >90 days following the initial bowel resection. Patients aged 17 years old and younger were included in the study if they were diagnosed with SBS at <2 years of life and excluded if they had extra-intestinal congenital anomalies or if they underwent liver or intestinal transplant. Stools were collected quarterly when possible, either from a diaper or a collection receptacle placed in the toilet prior to spontaneous stooling. Stool were immediately stored at −80°C until analyzed.

Clinical and demographic data were obtained from electronic medical records. Remnant short bowel length and presence of ileocecal valve were extracted from the surgical operative reports or intestinal rehabilitation clinic notes. Anthropomorphic measurements found in electronic medical records were obtained on the day of, or within a month of, stool collection. For participants under two years old, percentiles for weight and length were based on the World Health Organization (WHO) reference values after correcting for gestational age. For samples obtained when children were 2 years or older, the US Centers for Disease Control and Prevention (CDC) growth chart were used. Each course of intravenous antibiotic treatment was obtained from medication histories recorded during hospitalizations and oral antibiotic treatment obtained from intestinal rehabilitation clinic notes. This study was approved by Washington University’s IRB (201912067).

Preterm and term controls were selected from two previously published studies.^16,17^ All of the preterm and term samples and metadata were collected as part of the Neonatal Microbiome and Necrotizing Enterocolitis Study (P.I.T. and B.B.W.) or the St Louis Neonatal Microbiome Initiative (B.B.W. and P.I.T.) at Washington University School of Medicine and approved by the IRB (201105492 and 201104267, respectively). Samples were obtained from infants after parents provided informed consent. For each SBS patient, matching controls were selected based on similarity of age-distribution at sample collection points.

### Metagenomic DNA extraction

Metagenomic DNA was extracted from approximately 100 mg stool using the PowerSoil DNA Isolation Kit (Qiagen) following the manufacturer’s protocol, except that samples were mechanically lysed for two rounds of two minutes each using a Mini-Beadbeater-24 (Biospec Products) at 2,500 oscillations min^−1^. Metagenomic DNA was quantified using Qubit (Invitrogen) and stored at −20°C.

### BSI isolate collection and DNA extraction

Glycerol stocks of isolates cultured from SBS patients during hospitalizations for BSI at SLCH, St. Louis, Missouri, were used for this study. 1 μL from each specimen was plated to sheep’s blood agar plates (Hardy Diagnostics) using a 1 μL calibrated loop and incubated at 37°C for 24 hours. Genomic DNA was extracted using the Bacteremia DNA Extraction Kit (Qiagen) following the manufacturer’s protocol.

### Selective culturing of stools

Stool specimens from SBS patients with available BSI isolates were selected for selective culturing if the BSI causative species was as follows (1) detected in the metagenomic dataset and (2) was present at <5% relative abundance. The following media were used for selective enrichment of BSI causing pathogens: *E. coli –* MacConkey agar (Hardy Diagnostics), *Klebsiella* sp. – *Klebsiella* ChromoSelect Selective Agar (Millipore Sigma), *E. faecalis* – m-*Enterococcus* agar, modified (Millipore Sigma), *S. aureus* and *S. epidermidis* – Aureus ChromoSelect Agar (Millipore Sigma). Approximately 5 mg of stool material were plated onto the respective culture media, which were incubated for 24–48 hours (37°C). In cases of positive growth, plates were harvested and metagenomic DNA was extracted using the PowerSoilPro DNA Isolation Kit (Qiagen).

### Library preparation, sequencing, and quality filtering

Sequencing libraries from both isolate gDNA and metagenomic DNA were prepared using the Nextera kit (Illumina). Libraries were pooled and shotgun sequenced (2x150 bp) to a pre-determined depth of ~1.5 million reads (BSI isolates – 1.55 (1.29–1.77) [median IQR]) or ~5 million reads (fecal metagenomes – 5.17 (3.79–6.37) million reads [median (IQR)]) on the NextSeq 500 HighOutput platform (Illumina). For BSI-strain tracking, selected stools were sequenced to a coverage that facilitated ~50x genome coverage of the species of interest (8.54 (6.52–12.84) million reads [median (IQR)]), calculated based on relative abundance as determined from previous medium depth metagenomic sequencing. Selectively cultured isolates were sequenced to a depth of ~1.5 million reads (1.68 (1.47–2.21) million reads [median (IQR)]). The resulting reads were trimmed of adapters using Trimmomatic v.36 (parameters: LEADING:10 TRAILING:10 SLIDINGWINDOW:4:15 MINLEN:60) and depleted of human read contamination using DeconSeq v.4.3 (default parameters).^40,41^ Rarefaction analysis was performed for both taxonomic profiles (diversity/richness) and antibiotic resistance genes (diversity/richness), confirming appropriate coverage at the predetermined sequencing depth (Supplementary Figure 6). Rarefaction was used to establish appropriate coverage of the microbiome using the predetermined sequencing depth, and subsampled metagenomes were not used for analyses.

### Microbiome and statistical analysis

Quality filtered paired-end metagenomic reads from all cohorts were used to access microbial taxa relative abundance using MetaPhlAn2 v.2.6.0 (default parameters) and functional profiles of the intestinal microbiota using HumaNn2 v.2.8.1 (default parameters).^42,43^ Resistance gene abundance was determined using ShortBRED v.0.9.4 using marker sequences build on the CARD and NCBI AMR databases (default parameters).^44^ Statistical analysis and visualization were conducted in R v.3.6.3 using the ggplot2, ggpubr, VEGAN, BiodiversityR, ape, lme4, nlme, pheatmap, purr, dplyr, labdsv, reshape, ggpmisc, rowr, rsample, permute, rcompanion, multcomp, and MaAsLin2 packages.

α- and β-microbiota diversity were calculated using VEGAN. Repeat measures permutational analysis of variance (PERMANOVA) was implemented taking advantage of a previously developed custom script.^39^ Patient ID was included as a mandatory blocking factor in all repeat measure PERMANOVA. Variance explained by each clinical variable was calculated independently to avoid issues of variable ordering in the model formula. To account for repeat measures, permutations were performed blocked within participant for variables changing over time. For constant variables (e.g., length of bowel remaining, gestational age at birth), permutations were performed across participants. To determine variance explained by inter-individual variation, permutations were performed freely.

Maximum-likelihood generalized linear-mixed models (GLMMs) were implemented using the nlme package (lme function) or the MaAsLin2 package.^45^ Samples with missing data were excluded before analysis. All longitudinal GLMMs included patient ID as a mandatory random effect. To determine enriched/depleted taxa and functions, MaAsLin2 was run on relative abundance patterns of species or functions using default parameters. To assess between cohort differences for other features (e.g., α-diversity), all other model formulas followed the general structure:

feature ~ cohort*day of life + (1|Patient)

To assess the impact of clinical covariates on outcome of interest in the SBS cohort, variables were pre-screened for inclusion in a first (naïve) model as described.^46^ Following naïve model fitting, back-fitting was performed using the step() function. The best model from the set of back fitted models was selected based on model AIC values. *P*-value were corrected for multiple comparisons using Tukey’s method in the glht() function.

### BSI isolate strain-tracking

Draft genomes of BSI isolates were assembled using SPAdes v.3.11.0 (parameters: -k 21,33,55,77 -careful).^47^ The resulting scaffolds.fasta files were used for analysis. The quality of draft genomes were assessed by calculating assembly statistics using QUAST v5.0.2 and checkM v.1.0.13.^48,49^ Bowtie indices were constructed and reads from metagenomic samples and isolates were aligned to BSI isolate genomes using Bowtie2 v.2.3.5 (parameters: – very-sensitive – n-ceil 0,0.01).^50^ Pairwise core single nucleotide polymorphism (SNP) distances between BSI isolates and metagenomic strains were determined using the strainsifter workflow.^19^ Core SNPs were determined using mafft v. 7.471 (default parameters) and muscle v.3.8.1551 (default parameters) and phylogenetic trees were constructed using fasttree v.2.1.10 (parameter: -nt).

## Supplementary Material

Supplemental Material

## Data Availability

The sequencing data supporting these studies conclusions has been uploaded to NCBI SRA under the BioProject accession numbers PRJNA701982, PRJNA489090, PRJNA301903, PRJNA473126. All other supporting information is available from the corresponding author upon request.
