# Supplementary material for: Antibiotic-driven intestinal dysbiosis in pediatric short bowel syndrome is associated with persistently altered microbiome functions and gut-derived bloodstream infections
Source: Gut Microbes. 2021 Jul 15;13(1):1940792. doi: 10.1080/19490976.2021.1940792 (PMC8284144; doi:10.1080/19490976.2021.1940792)
Supplement: Supplemental Material [file KGMI_A_1940792_SM0926.zip › Supplementary information/210520_Supplemental_Material_Revised.docx]

**Supplemental Information for:**

**Antibiotic-driven intestinal dysbiosis in pediatric short bowel syndrome is associated with persistently altered microbiome functions and gut-derived bloodstream infections**

**Robert Thänert, Anna Thänert, Jocelyn Ou, Adam Bajinting, Carey-Ann D. Burnham, Holly J. Engelstad, Maria E Tecos, I. Malick Ndao, Carla Hall-Moore, Colleen Rouggly-Nickless, Mike A Carl, Deborah C. Rubin, Nicholas O. Davidson, Phillip I. Tarr, Barbara B. Warner, Gautam Dantas, Brad W Warner**

**Supplementary Figure 1 | Heatmap of significantly enriched and depleted species in the SBS vs term cohort as determined via MaAsLin2.** Relative abundance of species (columns) is depicted for samples (rows). Rows and columns are hierarchically clustered. Cohort identity of samples is given as color annotation: SBS (salmon), Term (teal).

**Supplementary Figure 2 | Heatmap of significantly enriched and depleted species in the SBS vs preterm cohort as determined via MaAsLin2.** Relative abundance of species (columns) is depicted for samples (rows). Rows and columns are hierarchically clustered. Cohort identity of samples is given as color annotation: SBS (salmon), Preterm (peach).

**Supplementary Figure 3 | Relative abundance and Shannon diversity of the gut microbiota for stool samples of patient SBS 05 by day of life.** Relative abundance of taxa is depicted as stacked bargraphs. Shannon diversity values for each sample are connected by a line. The history of antibiotic exposures is plotted as a schematic above the graph.

**Supplementary Figure 4 | Resistome diversity and richness by year of life.** (A) Shannon diversity of the resistome of SBS patients (salmon), preterm (purple) and term controls (teal) by year of life (*n*=159). Loess regression lines with 95% confidence interval shading are drawn. (B) ARG richness of the resistome of SBS patients (salmon), preterm (purple) and term controls (teal) by year of life (*n*=159). Loess regression lines with 95% confidence interval shading are drawn.

**Supplementary Figure 5 | Relative ARG class abundance by year of life.** Relative abundance of (A) Resistance Modulator, (B) Efflux Pump, (C) Lincosamide, and (D) Fosfomycin ARGs in SBS patients (salmon), preterm (purple) and term controls (teal) by year of life (*n*=159). Loess regression lines with 95% confidence interval shading are drawn. All *P-*values are two-tailed, from longitudinal maximum-likelihood GLMMs Tukey-adjusted for multiple comparisons.

**Supplementary Figure 6 | Rarefaction analysis.** Boxplots depicting taxonomic (left) and resistance gene (‘Resistome’, right) diversity (left panel) and richness (right panel) for SBS (top), term (mid) and preterm (bottom) stool metagenomes at rarefied sequencing depths. Significant differences between rarefied bins are indicated (*P*<0.05, ANOVA Tukey post-hoc corrected for multiple comparisons).
